# Tanshinone IIA alleviates ovalbumin-induced allergic rhinitis symptoms by inhibiting Th2 cytokine production and mast cell histamine release in mice

**DOI:** 10.1080/13880209.2022.2034894

**Published:** 2022-02-15

**Authors:** Qing Chen, Liping Shao, Yong Li, Mian Dai, He Liu, Nan Xiang, Hui Chen

**Affiliations:** Department of Otorhinolaryngology, Chengdu Integrated TCM and Western Medicine Hospital, Chengdu, China

**Keywords:** Inflammation, OVA-specific IgE/IgG1, nose rubs

## Abstract

**Context:**

Studies have shown that tanshinone IIA (TIIA) has an anti-inflammatory effect, but the effect on allergic rhinitis (AR) is unclear.

**Objective:**

In this study, we explore the effect of TIIA on AR.

**Materials and methods:**

AR mice model was established by the intraperitoneal (ip) injection of 50 μg ovalbumin (OVA). AR mice in the dose tested groups were treated with TIIA (10 mg/kg/d, ip) or dexamethasone (Dex) (2.5 mg/kg/d, oral). The number of nasal rubbing in mice was counted. Inflammatory, goblet and mast cells in nasal mucosal tissue were detected. The contents of histamine, OVA-immunoglobulin E (IgE), OVA-immunoglobulin G1 (IgG1), tumour necrosis factor-α (TNF-α), interleukin-4 (IL-4), IL-5, interferon-γ (IFN-γ) and IL-12 in nasal lavage fluid (NALF) or serum were measured. Human mast cells (HMC-1) were treated with C48/80 to release histamine or TIIA for therapeutic effect, and the cell viability, histamine content and mast cell degranulation were examined.

**Results:**

OVA promoted the number of nasal rubbings in mice (78 times/10 min, *p*< 0.001), increased the inflammatory, goblet and mast cells in nasal mucosal tissue, and significantly (*p*< 0.001) elevated the levels of histamine (120 ng/mL), OVA-IgE (2 pg/mL), OVA-IgG1 (90 ng/mL), TNF-α (2.3 pg/mL), IL-4 (150 pg/mL) and IL-5 (65 pg/mL) in serum or NALF of OVA-induced AR mice. However, both TIIA and Dex inhibited the effect of OVA on AR mice. Besides, TIIA reversed the promotion of histamine release (30%) and mast cell degranulation induced by C48/80.

**Discussion and conclusions:**

TIIA alleviates OVA-induced AR symptoms in AR mice, and may be applied as a therapeutic drug for patients with Th2-, or mast cell-allergic disorders.

## Introduction

Allergic rhinitis (AR) is a chronic inflammatory disease of the upper respiratory tract, with recurrent sneezing and nasal itching as the main clinical features, which reduces the life quality of patients and has become a global public health problem (Bousquet et al. 2019; Eiringhaus et al. 2019). Relevant surveys have shown that AR is one of the risk factors that may induce bronchial asthma, and the two diseases coexist in many patients; active treatment of AR is also one of the effective measures to prevent or reduce bronchial asthma (Bousquet et al. 2012; Price et al. 2020). Therefore, it is of great practical significance to investigate the pathogenesis of AR and further explore effective treatment.

Studies have demonstrated that AR is an allergic and chronic non-specific inflammatory reaction mediated by the body’s immune factors, especially the immunoglobulin E (IgE) which could modulate non-infectious inflammation on the rhinitis membrane (Bernstein et al. 2016; Bayar Muluk et al. 2019). Histological features of AR include enhanced production of inflammatory cytokines and eosinophil infiltration, which are often accompanied by Th2 cytokine production and mast cell histamine release (Bui et al. 2017). There are many methods to treat AR, mainly consisting of four aspects: drug therapy, immunotherapy, environmental control and patient education (Cobanoğlu et al. 2013; Jia et al. 2017). A study in the United States has revealed that recent advances in the treatment of AR include the use of nasal antihistamines and nasal steroids, and drug therapy is the main pillar in the treatment of AR (Hoyte and Nelson 2018). At present, the main drugs for the treatment of AR in clinical practice are: antihistamines, glucocorticoids, anti-leukotrienes and mast cell stabilizers (Wise et al. 2018). These drugs could reduce the symptoms of AR with different mechanisms of action, some of which, however, may cause other side effects on the body (Mandhane et al. 2011).

With the continuous development of modern pharmacological research, a variety of Chinese herbal monomer components have been confirmed to be effective in the treatment of AR, such as quercetin (Sagit et al. 2017). Tanshinone IIA (TIIA) is one of the fat-soluble active components with clear molecular structure (C_19_H_18_O_3_) extracted from the roots of medicine *Salvia miltiorrhiza* Bunge (Lamiaceae), locally known as ‘*Danshen*’ in Chinese herbal medicine, which is a monomer that has been well studied in medicine because of its stable active ingredients (Li C et al. 2016; Li Q et al. 2016). It has been confirmed that TIIA not only has anti-tumour and oxygen free radical scavenging effects, but also plays an important role in the anti-inflammatory field (Cheng et al. 2019). Several previous studies have discovered that TIIA reduces lipopolysaccharide (LPS)-induced acute lung injury by inhibiting inflammation and apoptosis in mice. TIIA can inhibit the production of inflammatory factors such as tumour necrosis factor-α (TNF-α) in RAW264.7 macrophages. Besides, TIIA reduces IgE-mediated local and systemic allergic reactions in mice, which is associated with the inhibition of mast cell activation (Fan et al. 2009; Xu et al. 2015; Li X et al. 2018). However, the relevant biological mechanisms regarding the action of TIIA in AR are still unclear.

In this study, we observed the effect of TIIA on relieving allergic symptoms through a mouse model of AR induced by ovalbumin (OVA), and also detected the contents of inflammation-related factors in OVA-induced AR mice, hoping to provide a new direction for the research and development of AR drugs.

## Materials and methods

### Ethics statement

All animal experiments were performed in accordance with the guidelines of the China Council on Animal Care and Use. This study was approved by the Committee of Experimental Animals of Chengdu Integrated TCM and Western Medicine Hospital (JX2019121304). Every effort was made to minimize pain and discomfort to the animals. The animal experiments were performed in Chengdu Integrated TCM and Western Medicine Hospital.

### Animal model and grouping

**Figure 1.**
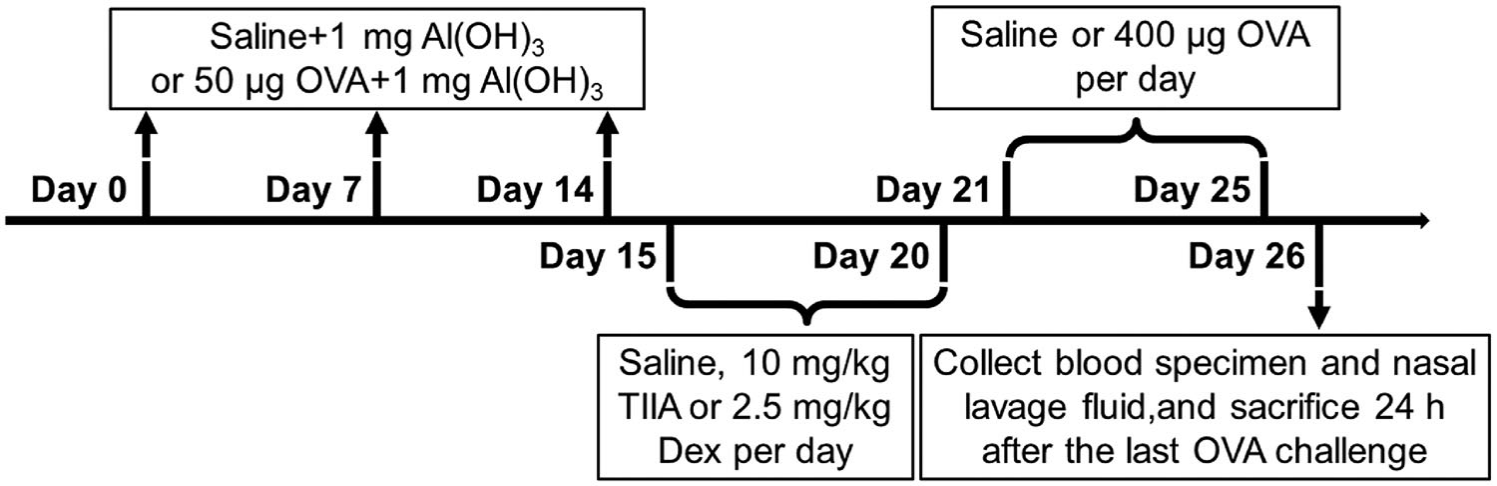
The experimental procedures for the mouse model of AR.

Forty 6-week-old male BALB/c mice (weighed 25–30 g) were purchased from Jiangsu ALF Biotechnology Co., Ltd. (Nanjing, China). As reported in relevant studies, all mice were housed in a level specific pathogen free (SPF) animal room at room temperature 22–24 °C with free access to water and a 12 h light/dark cycle (Bui et al. 2017). The experimental mice were randomly divided into four groups (10 mice in each group): (1) control group, (2) OVA (O1641, Sigma-Aldrich, St. Louis, MO) group, (3) OVA + TIIA (SML2517, Sigma-Aldrich, St. Louis, MO) group, (4) OVA + dexamethasone (Dex; D4902, Sigma-Aldrich, St. Louis, MO) group. The experimental process of AR mouse model is depicted in Figure 1. Three times intraperitoneal (ip) injections of OVA were given on day(s) 0, 7 and 14. Specifically, in control group, the mice were treated with saline combined 1 mg Al(OH)_3_ (239186, Sigma-Aldrich, St. Louis, MO); whereas in OVA, OVA + TIIA and OVA + Dex groups, the mice were administrated with 50 μL OVA combined 1 mg AI(OH)_3_. From days 15 to 20, the following treatments were performed for mice in each group: the mice were treated with saline in control group and OVA group; the mice were given 10 mg/kg TIIA (SML2517, Sigma-Aldrich, St. Louis, MO) in OVA + TIIA group; and the mice were administrated with 2.5 mg/kg Dex (D4902, Sigma-Aldrich, St. Louis, MO), per day in the OVA + Dex group. From days 21 to 25, the mice were treated once daily with saline in the control group; and mice were given once daily with 400 μg OVA in OVA, OVA + TIIA and OVA + Dex groups. Twenty-four hours after the last OVA administration (day 26), mice were sacrificed by cervical dislocation after anaesthetic treatment with acepromazine (0.75 mg/kg; A7111, Sigma-Aldrich, St. Louis, MO). Following the indicated treatment, their blood specimen, nasal mucosal tissue and nasal lavage fluid (NALF) were collected.

### Nasal allergic symptoms

After the last OVA challenge, the mice were placed in a cage for observation of nose scratching for 10 min, and the scratching number was recorded separately.

### Haematoxylin and eosin (H&E) staining

The collected nasal mucosa tissues of mice were fixed in 10% neutral formalin (HT501128, Sigma-Aldrich, St. Louis, MO) for 24 h, and then embedded in paraffin (P3558, Sigma-Aldrich, St. Louis, MO). Next, the tissues were made into sections using paraffin slicer (E0972, Beyotime, Shanghai, China). Subsequently, the sections were immersed in xylenes (247642, Sigma-Aldrich, St. Louis, MO) for deparaffinization. After that, the tissue samples were placed in different gradients of ethanol prepared with 100% ethanol (E7023, Sigma-Aldrich, St. Louis, MO) and double distilled water for sample hydration. Later, the tissue sections were soaked and washed three times for 3 min using phosphate buffer saline solution (PBS; C0221A, Beyotime, Shanghai, China). After being stained thoroughly with haematoxylin solution (C0107, Beyotime, Shanghai, China) for 10 min and eosin solution (C0109, Beyotime, Shanghai, China) for 3 min, the sections were soaked with xylene twice for 4 min each time, and then the tissue samples were sealed with neutral resin. Finally, the sections were observed and photographed under an optical microscope (DMi8, Leica, Wetzlar, Germany) at a magnification of ×200.

### Periodic acid-Schiff (PAS) staining

The nasal mucosal tissues of mice were fixed in 10% neutral formalin for 24 h, routinely paraffin-embedded and sectioned. After routine deparaffinization to water, sections were oxidized with 1% sodium periodate aqueous solution (363642, Sigma-Aldrich, St. Louis, MO) for 10 min and washed three times with distilled water for 2 min each time. Then, the sections were stained with Schiff solution (S5133, Sigma-Aldrich, St. Louis, MO) for 20 min, followed by 10-min rinsing with distilled water for differentiation. After 10 s of staining with haematoxylin, the sections were differentiated by 1% hydrochloric acid in ethanol for 20 s, then rinsed with tap water and dehydrated with different gradients of ethanol, followed by being cleared using xylene and mounted with neutral gum (G9752, Sigma-Aldrich, St. Louis, MO). Finally, the sections were observed and photographed under an optical microscope (magnification ×200).

### Giemsa staining

Paraffin-embedded sections were prepared with the harvested mouse nasal mucosal tissues. Next, the sections were deparaffinized by soaking in xylene for 5–10 min. Afterwards, the sections were switched to fresh xylene, and deparaffinized for another 5–10 min. Subsequently, the sections were dehydrated as follows: immersed in absolute ethanol for 5 min, 90% ethanol for 2 min, 80% ethanol for 2 min and 70% ethanol for 2 min. Appropriate amount of prepared modified Giemsa staining working solution (C0131, Beyotime, Shanghai, China) was added to the sections for 45 min of staining, and the images of sections were observed and photographed under an optical microscope at the magnification of ×200.

### Measurement of OVA-specific IgE and IgG1

Mice serum was collected, and the concentrations of OVA-IgE and OVA-immunoglobulin G1 (IgG1) in mice serum were measured according to the instructions of mice OVA-IgE enzyme-linked immunosorbent assay (ELISA) kit (F10731, Westang, Shanghai, China) and mice OVA-IgG1 ELISA kit (3013, Chondrex, Woodinville, WA), respectively. Anti-mouse IgE monoclonal antibody (100 mL) was added to each well of 96-well ELISA plates, followed by incubation at 4 °C overnight. The plate was washed with diluted wash buffer after the coating solution was discarded. With the supplement of 100 μL blocking buffer to each well, the plate was maintained at room temperature for 1 h. After the solution was discarded, each well of 96-well ELISA plates was added with the prepared standard sample for 90 min of incubation at room temperature. Following the addition of 100 μL dissolved biotinylated OVA to each well, the plate was cultured at room temperature for another 90 min. Subsequently, the 96-well ELISA plates were washed, each well of which was supplemented with 100 μL TMB solution, followed by the culture in the dark at room temperature for 30 min. Finally, 50 μL stop solution was put to each well of 96-well ELISA plates. The optical density (OD) value was measured at a wavelength of 450 nm with a microplate reader (SpectraMax iD3, Molecular Devices, Silicon Valley, CA).

### Assessment of cytokines of Th1 (IL-12 and IFN-γ) and Th2 (TNF-α, IL-4 and IL-5)

Specimens of NALF were removed from the refrigerator at −70 °C and dissolved at room temperature. Subsequently, the levels of IL-12, interferon-γ (IFN-γ), TNF-α, interleukin-4 (IL-4) and IL-5 were measured with ELISA kits (ab282874, ab119531, ab208348, ab100710 and ab204523, respectively) brought from Abcam (Cambridge, UK), according to the manufacturer's instructions. The absorbance at 450 nm was measured with a microplate reader.

### Cell line culture

Normal human mast cell line HMC-1 (JCRB0166, BioVector NTCC, Beijing, China) was cultured in Dulbecco's modified Eagle's medium (DMEM; D0819, Sigma-Aldrich, St. Louis, MO) containing 10% foetal bovine serum (FBS; C0227, Beyotime, Shanghai, China) at 37 °C with 5% CO_2_.

### Methyl tetrazolium (MTT) assay

The cells in logarithmic phase were collected, and then added to each well of the 96-well plate after the cell density was adjusted to 2 × 10^3^ cells/well. Later, 5 μmol/L TIIA and 10 μmol/L TIIA were put into each well, and the plate was cultured in a cell incubator at 37 °C containing 5% CO_2_ for 1 h. Next, 10 μL MTT solution (C11019-2, RIBOBIO, Guangzhou, China) was supplemented to each well, followed by the culture at 37 °C for 4 h. Finally, with an addition of 100 μL dimethyl sulphoxide (DMSO; ST038, Beyotime, Shanghai, China) to each well, the plate was shaken on a shaker at low speed for 2 min. Eventually, the absorbance of each well was measured using a microplate reader with an excitation wavelength of 570 nm.

### Measurement of histamine

Histamine levels from serum of mice were determined using *o*-phthalaldehyde spectrofluorometric analysis (Moon et al. 2018). The fluorescent intensity was measured at a wavelength of 440 nm (excitation at 360 nm) in a spectrofluorometer (RF-6000, Shimadzu, Kyoto, Japan).

Cells were then centrifuged at 4 °C, and the culture medium (cell-free supernatants) and total suspensions were collected and analysed for histamine content by Histamine levels in cells using histamine ELISA kit (ab213975, Abcam, Cambridge, UK). According to the instructions of the experimental procedures, the frozen samples were taken out and slowly thawed at room temperature. The assay buffer was added into the well binding for 0 ng/mL standard (Bo) or non-specific binding (NSB) well. Besides, 100 μL of standard #1 to #5 were supplemented into appropriate wells. Likewise, 100 μL of the samples were put into the appropriate wells. Afterwards, 50 mol/L of 1× histamine tracer and 50 mol/L 1× histamine antibody were added. After the incubation at room temperature for 1 h, each well was washed twice with washing solution and then added with 200 μL of SA-HR and 200 μL of TMB substrate solution. After each well was added with 50 μL of the stop solution, OD value of each well was measured with a microplate reader at a wavelength of 450 nm.

The concentration of histamine release from mast cells was determined by histamine test kit. Compound 48/80 (C48/80; C2313, Sigma-Aldrich, St. Louis, MO) can be used to facilitate histamine release (Won Jung et al. 2012). The collected human mast cell line HMC-1 in logarithmic growth phase was divided into four groups: (1) control group, (2) C48/80 group, (3) C48/80 + 5 μmol/L TIIA (TIIA-5) group and (4) C48/80 + 10 μmol/L TIIA (TIIA-10) group. Cells in each group (except for control group) were pre-treated using 0.5 μg/mL C48/80 at 37 °C for 1 h. Then, the cells in C48/80 + 5 μmol/L TIIA (TIIA-5) and C48/80 + 10 μmol/L TIIA (TIIA-10) groups, were treated with C48/80 and TIIA at different concentrations at 37 °C for 30 min, respectively. Finally, the culture supernatant was collected after centrifugation at 400×*g* for 5 min at 4 °C. Same as the above experimental procedures, mast cell histamine release was detected by histamine ELISA kit. The OD value of each well was measured with a microplate reader at a wavelength of 450 nm. Histamine release was calculated as the percent of total (cellular + extracellular) histamine.

### Detection of mast cell degranulation

As described above, mast cell line HMC-1 was collected and divided into the following four groups: (1) control group, (2) C48/80 group, (3) C48/80 + 5 μmol/L TIIA (TIIA-5) group and (4) C48/80 + 10 μmol/L TIIA (TIIA-10) group. Cells in different groups were treated with 0.5 μg/mL C48/80 and different concentrations of TIIA. After incubation, the cells were fixed with 4% paraformaldehyde (P0099, Beyotime, Shanghai, China) for 25 min, and then stained with toluidine blue (T3260, Sigma-Aldrich, St. Louis, MO) for 1–2 min. Finally, mast cell degranulation was observed under an optical microscope.

### Statistical analysis

GraphPad Prism 8.0 (GraphPad Software, La Jolla, CA) was used for statistical analysis. Measurement data were expressed as mean ± standard deviation (m±*s*). One-way analysis of variance was utilized for comparison among multiple groups. Tukey’s test was employed for pairwise comparison. *p*< 0.05 was considered as statistically significant.

## Results

### OVA promoted the number of nasal rubbing in mice, while both TIIA and Dex inhibited the number of nasal rubbing in OVA-induced AR mice

**Figure 2.**
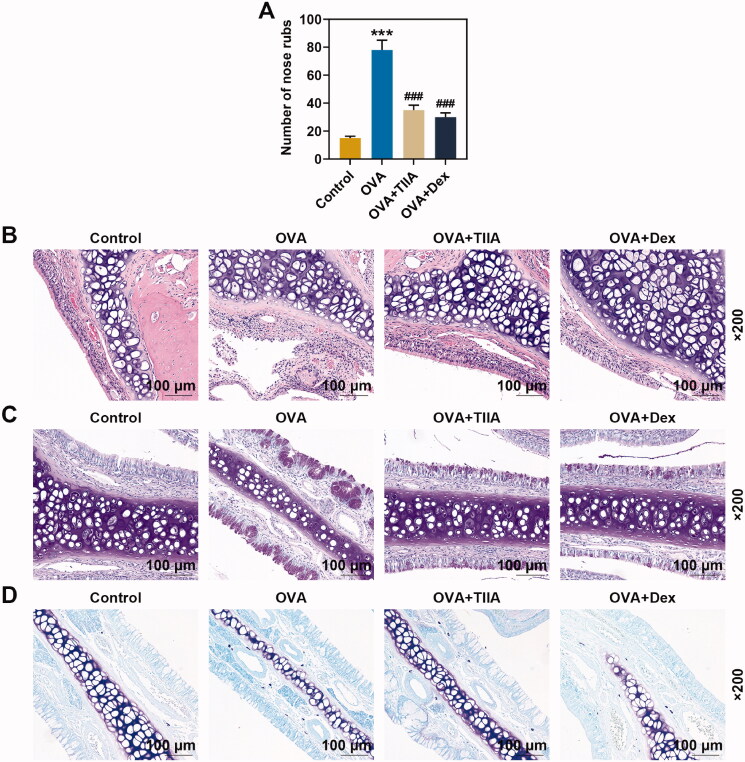
OVA increased the number of nasal rubbing in mice, inflammatory response, goblet cell and mast cell in nasal mucosal tissue of OVA-induced AR mice, while both TIIA and Dex inhibited the effect of OVA. (A) The nasal rubbing of mice was observed and its number was counted. (B) Inflammatory cells in nasal mucosal tissue were detected by H&E staining. (C) Goblet cells in nasal mucosal tissue were determined by PAS staining. (D) Mast cells in nasal mucosal tissue were observed by Giemsa staining. ***p < 0.005, ^###^*p* < 0.005, * vs. control; ^#^ vs. OVA.

First, we established OVA-induced AR mouse model according to the process shown in Figure 1. The nasal scratching in mice was observed for 10 min, and the recorded data showed that the number of sneezing in mice with AR was significantly increased in OVA group as compared with that in control group. Meanwhile, the effect of OVA was markedly suppressed in Dex group and TIIA group, when compared with that in OVA group (Figure 2(A), *p*< 0.001). Therefore, we speculated that Dex and TIIA could also slow the symptoms in OVA-induced AR mice, but their effects still needed to be further demonstrated.

### OVA increased the inflammatory response, goblet cell and mast cell in nasal mucosal tissue of OVA-induced AR mice, while both TIIA and Dex inhibited the effect of OVA

H&E staining revealed that the nasal mucosa of mice in the control group was normal, without inflammatory cell infiltration (Figure 2(B)). Compared with that in control group, a large number of inflammatory cell infiltration appeared around the nasal mucosa of mice in the OVA group, and the inflammatory infiltration in the OVA + TIIA group and OVA + Dex group was improved compared with that in the OVA group. Mice nasal mucosa was stained with PAS to visualize goblet cells. As delineated in Figure 2(C), the cytoplasm of the goblet cells was stained violaceous. There were only very few scattered goblet cells in the mucosal epithelium of normal control mice. However, compared with the control group, not only the number of goblet cells in the nasal mucosa of mice was remarkably increased, but also the cytoplasm in the nasal mucosa of mice was significantly proliferated in the OVA group. Additionally, the goblet cells in the hypertrophic part showed a typical high foot goblet shape, and were obviously reduced in the nasal mucosa of mice treated with TIIA and Dex, respectively, as compared with those in the nasal mucosa of mice treated with OVA. Giemsa staining was applied for the detection of mast cells, and the result indicated that mast cells in OVA group were increased dramatically when compared with those in the control group, while after TIIA or DEX treatment, the number of mast cell was decreased compared with that in the OVA group (Figure 2(D)). Our results preliminarily suggested that TIIA exerted a positive effect on the treatment of AR mice, but its mechanism of effect still required further exploration.

### OVA increased the levels of histamine, OVA-IgE and OVA-IgG1 in the serum of OVA-induced AR mice, while both TIIA and Dex inhibited the effects of OVA

**Figure 3.**
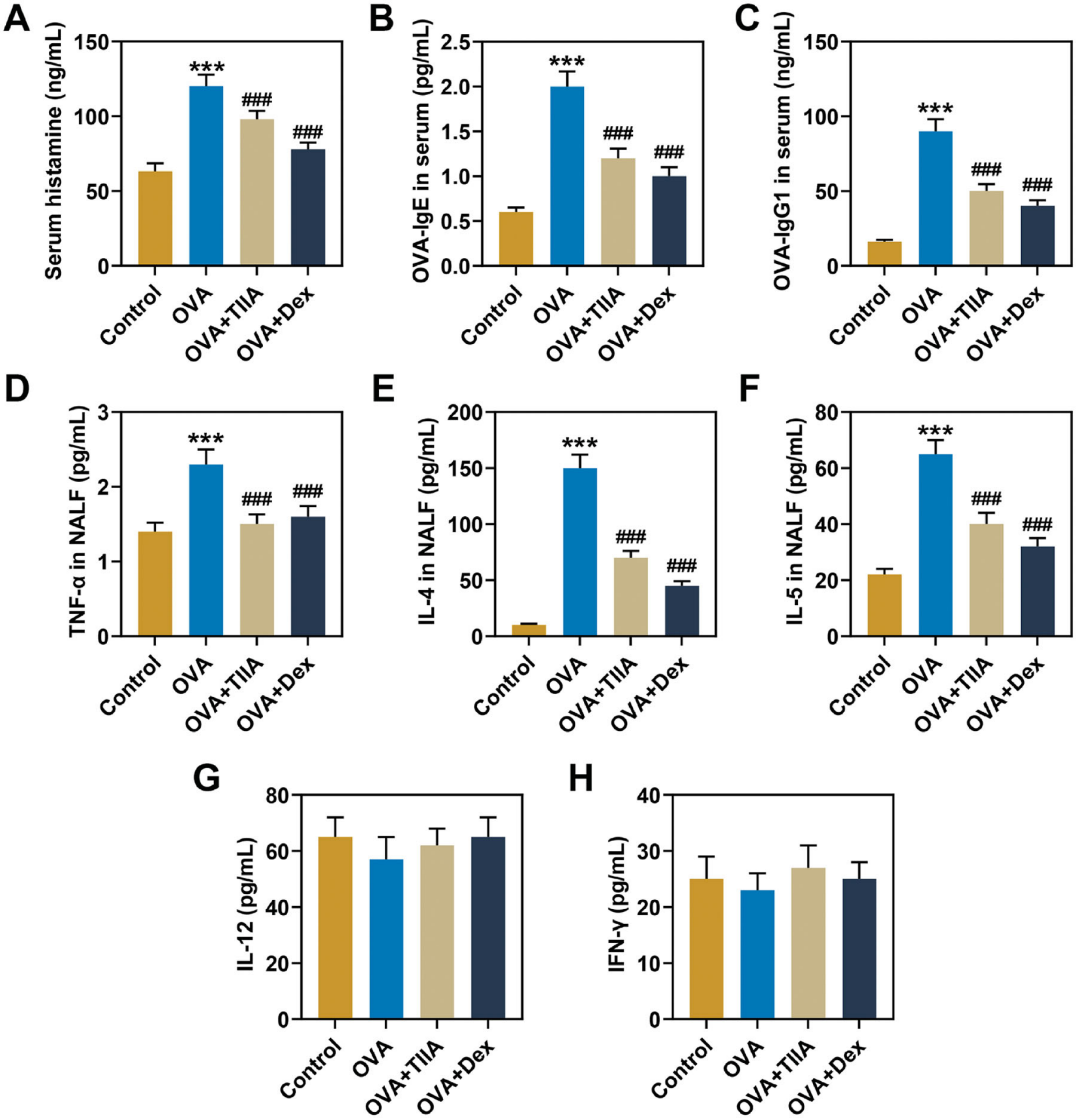
The effects of OVA and Dex on the levels of OVA-IgE, OVA-IgG1, TNF-α, IL-4, IL-5, IL-12 and IFN-γ in the serum and NALF of OVA-induced AR mice. (A) The contents of histamine in the serum were measured by *o*-phthalaldehyde spectrofluorometric method. (B, C) The contents of OVA-IgE and OVA-IgG1 in the serum were detected by ELISA. (D–H) The contents of TNF-α, IL-4, IL-5, IL-12 and IFN-γ in the NALF were tested by ELISA. ***p < 0.005, ^###^*p* < 0.005, * vs. control; ^#^ vs. OVA.

The experimental results displayed that the histamine content of mice serum in the OVA group was evidently increased compared with that in the control group (*p*< 0.001), while the histamine content in the OVA + TIIA group and OVA + Dex group was clearly reduced as compared with that in the OVA group (Figure 3(A), *p*< 0.001). Similarly, the results of ELISA showed that the levels of OVA-IgE and OVA-IgG1 in the OVA group were observably elevated in comparison with those in the control group (*p*< 0.001), while the levels of OVA-IgE and OVA-IgG in the OVA + TIIA group and OVA + Dex group were markedly reduced when compared with those in the OVA group (Figure 3(B,C), *p*< 0.001). In the present study, we evaluated that OVA promoted the levels of histamine, OVA-IgE and OVA-IgG1 in the mice serum of the OVA group, while both TIIA and Dex inhibited the effects of OVA in AR mice.

### The effects of TIIA and Dex on the production of Th1/Th2 cytokines in the NALF of OVA-induced AR mice

The levels of TNF-α, IL-4, IL-5, IL-12 and IFN-γ in NALF were measured by ELISA, and the results showed that, compared with those in the control group, the levels of TNF-α, IL-4 and IL-5 in mice of OVA group were observably increased (*p*< 0.001), while the contents of TNF-α, IL-4 and IL-5 in mice of the OVA + TIIA group and OVA + Dex group were significantly reduced as compared with those in mice of OVA group (Figure 3(D–F), *p*< 0.001). Additionally, the levels of IL-12 and IFN-γ did not change significantly in mice of each group (Figure 3(G,H)). These results demonstrated that, similar to Dex, TIIA evidently inhibited the contents of TNF-α, IL-4 and IL-5 in NALF of OVA-induced AR mice.

### TIIA at 5 and 10 μmol/L had no effect on the viability of human mast cells, and reversed the promotion of histamine release and mast cell degranulation induced by C48/80

**Figure 4.**
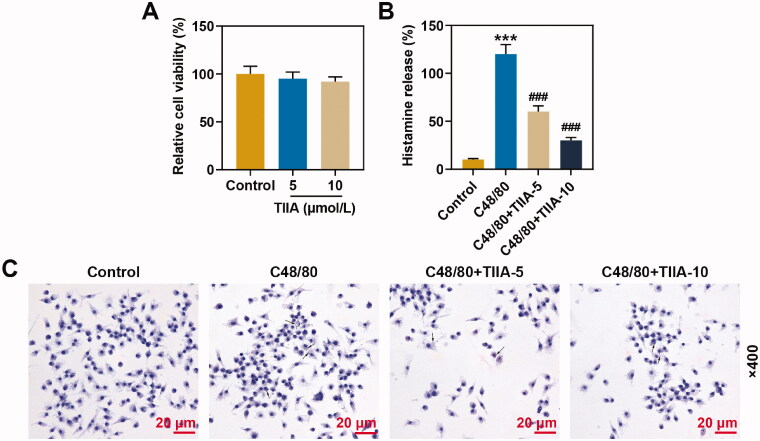
TIIA at 5 and 10 μmol/L had no effect on the viability of human mast cells, and reversed the promotion of histamine release and mast cell degranulation induced by C48/80. (A) The viability of human mast cell line HMC-1 cells after being treated with TIIA was examined by MTT assay. (B) Histamine content of human mast cell line HMC-1 cells after treatment with C48/80 and TIIA in culture medium (cell-free supernatants) and total suspensions was determined by ELISA. (C) The degranulation of human mast cell line HMC-1 cells after being treated with C48/80 and TIIA was detected by toluidine blue staining. ***p < 0.005, ^###^*p* < 0.005, * vs. control; ^#^ vs. OVA. TIIA: tanshinone IIA; MTT: methyl tetrazolium; ELISA: enzyme-linked immunosorbent assay; C48/80: compound 48/80.

The results of cell viability assay suggested that TIIA had no significant effect on the viability of human mast cell line HMC-1 (Figure 4(A)). ELISA exhibited that histamine release was promoted in C48/80 group as compared with that in the control group, while the histamine content in the C48/80 + TIIA-5 group and C48/80 + TIIA-10 group was memorably reduced, when compared with that in the C48/80 group (Figure 4(B), *p*< 0.001). Experiments have mirrored that TIIA overturned the promotion of histamine release induced by C48/80. Similarly, C48/80 facilitated the mast cell degranulation when compared with that in the control group, while the mast cell degranulation on the C48/80 + TIIA-5 group and C48/80 + TIIA-10 group was prominently reduced in comparison with that in the C48/80 group (Figure 4(C)). These findings revealed that TIIA offset the promotion of histamine release and mast cell degranulation induced by C48/80.

## Discussion

AR is a common and frequent chronic inflammatory disease mediated by IgE after specific individual exposure to allergens (Eifan and Durham 2016). At present, drug treatment is the main method for AR, and the drugs widely used in clinical practice are antihistamines, corticosteroids and anti-leukotrienes, as well as mast cell stabilizers (Ricketti et al. 2017; Wise et al. 2018). However, prior research has signified that these drugs could cause other side effects on the body. For instance, the antihistamines with a positive therapeutic effect on sneezing, but without significant blocking effect, together with the first-generation antihistamines associated with sedation and psychomotor activity (Mygind 2014). In addition, long-term use of inhaled steroids is often accompanied by symptoms such as throat irritation, dry nose and headache, especially when large doses are used, drowsiness may be elicited (Bui et al. 2019; Kawauchi et al. 2019). Therefore, it is necessary to explore new drugs for the treatment of AR.

Establishing a mouse model of AR using OVA (as an allergen) is one of the methods commonly used to study AR, and this model has the advantages of simple operation and good repeatability (Gao et al. 2017; Liu et al. 2018). Dex is a drug commonly used in clinical practice to treat AR and can improve the symptoms of patients with AR (Liang et al. 2020). In this study, Dex was used as a positive control drug, and we observed improvement in nasal symptoms in mice treated with Dex. At the same time, we found that treatment with TIIA also alleviated nasal symptoms in mice with AR. Therefore, we speculated that TIIA and Dex had similar effects that could relieve symptoms in OVA-induced AR mice, but their specific effects still needed further investigation.

Previous studies have indicated that inflammatory cells refer to the cells involved in the inflammatory response in the human body; mast cells play important roles in type 1 hypersensitivity and are involved in the progression of AR; goblet cells are also involved in the progression of AR; and the inflammatory response of the nasal mucosa of patients with AR includes IgE-mediated mast cell response and goblet cell response, etc. (Broide 2010; Pawankar et al. 2011; Zhang et al. 2016; Li Q et al. 2018). Consistent with the results of our experiment in the mucosal tissue of OVA-induced AR mice, several prior researches have signified a large number of inflammatory cell infiltration, goblet cell hyperplasia and mast cell hyperplasia in mucosal tissue of AR mice (Li Q et al. 2018; Fan et al. 2019). In this research, in order to investigate the effect of TIIA on AR, AR model mice were treated with TIIA and Dex. The results profiled that both TIIA and Dex significantly inhibited inflammatory cell infiltration, mast cell proliferation and goblet cell proliferation in OVA-induced AR mice tissue, revealing that TIIA and Dex had similar effects that could relieve the inflammatory cell infiltration, mast cell hyperplasia and goblet cell hyperplasia in AR mice, but their mechanisms of action still required further exploration.

Histamine plays a major role in AR, and the release of histamine into the nasal mucosa can be facilitated by allergen induction in individuals (Taylor-Clark 2010). Allergic reactions are mainly caused by the production and action of IgE, IgG1 and other antibodies (Fan et al. 2019). Studies have demonstrated that according to different CD4^+^T, secreted factors of lymphocytes can be divided into Th1 cells and Th2 cells, among which Th2 cells could promote the synthesis of IgE in allergic reactions mainly by secreting IL-4, IL-5, TNF-α and other Th2 cytokines, with AR as the result of the predominance of Th2 cytokines over Th1 cytokines (Xu et al. 2018; Kim et al. 2019). In OVA-sensitized and challenged mice AR models, OVA-IgE in serum and Th2 cytokines such as IL-4 and IL-5 in NALF are increased (Lee et al. 2016; Suzuki et al. 2017). In this experiment, we also found that the contents of histamine, OVA-IgE and OVA-IgG1 in the serum and levels of TNF-α, IL-4 and IL-5 in NALF of OVA-induced AR mice were pronouncedly increased. TIIA has many biological activities, which can attenuate acute lung injury by inhibiting inflammation and apoptosis in mice, and reduce IgE-mediated local and systemic allergic reactions in mice (Li X et al. 2018). In this study, we also discovered that TIIA significantly decreased the contents of histamine, OVA-IgE, OVA-IgG1, TNF-α, IL-4 and IL-5 of OVA-induced AR mice. Accordingly, and intriguingly, in the present study, we demonstrated that in OVA-induced AR mice, TIIA inhibited the production of Th2 cytokines and the release of histamine from mast cells.

To further validate the effect of TIIA on mast cells *in vitro*, we cultured the human mast cell line HMC-1 and selected two different concentrations (5 and 10 μmol/L) of TIIA to treat the cells. TIIA is a natural plant extract component, and studies have revealed that TIIA is a very safe drug that can promote apoptosis of ovarian cancer cell line and protect H9c2 cells from oxidative stress-induced cell death (Gu et al. 2016; Li N et al. 2018). In this experiment, TIIA at 5 and 10 μmol/L had no effect on the cell viability of mast cell line HMC-1, and TIIA overtly reversed the promotion of histamine release and mast cell degranulation induced by C48/80.

Therefore, our experiments indicated that TIIA alleviated OVA-induced AR symptoms by blocking Th2 cytokine production and mast cell histamine release in mice AR model, and in human cell experiments, TIIA also inhibited histamine release and mast cell degranulation. This experiment provided a theoretical basis for the research and development of AR drugs.

## Data Availability

The analysed data sets generated during the study are available from the corresponding author on reasonable request.
